# Genetic Effects of Grain Quality Enhancement in *Indica* Hybrid Rice: Insights for Molecular Design Breeding

**DOI:** 10.1186/s12284-024-00719-7

**Published:** 2024-06-14

**Authors:** Ju Gao, Lijun Gao, Weiwei Chen, Juan Huang, Dongjin Qing, Yinghua Pan, Chonglie Ma, Hao Wu, Weiyong Zhou, Jingcheng Li, Xinghai Yang, Gaoxing Dai, Guofu Deng

**Affiliations:** 1 Guangxi Crop Genetic Improvement and Biotechnology Laboratory, Guangxi Academy of Agricultural Sciences, Nanning, Guangxi 530007 China; 2 Rice Research Institute, Guangxi Key Laboratory of Rice Genetics and Breeding, Guangxi Academy of Agricultural Sciences, Nanning, Guangxi 530007 China

**Keywords:** High-quality improvement, Molecular basis, Molecular breeding, MAS, *Indica* hybrid rice

## Abstract

Improving rice quality remains a crucial breeding objective, second only to enhancing yield, yet progress in quality improvement lags behind yield. The high temperature and ripening conditions in Southern China often result in poor rice quality, impacting hybrid rice production and utilization. Therefore, to address this challenge, analyzing the molecular basis of high-quality traits is essential for molecular design breeding of high-quality hybrid rice varieties. In this study, we investigated the molecular basis of grain shape, amylose content, gel consistency, gelatinization temperature, and aroma, which influence rice quality. We discovered that quality related alleles *gs3*, *GW7*^*TFA*^, *gw8*, *chalk5*, *Wx*^*b*^, *ALK*^*TT*^, and *fgr* can enhance rice quality when applied in breeding programs. Polymerization of *gs3*, *GW7*^*TFA*^, *gw8*, and *chalk5* genes improves rice appearance quality. The *gs3* and *GW7*^*TFA*^ allele polymerization increasing the grain’s length-width ratio, adding the aggregation of *gw8* allele can further reducing grain width. The *chalk5* gene regulates low chalkiness, but low correlation to chalkiness was exhibited with grain widths below 2.0 mm, with minimal differences between *Chalk5* and *chalk5* alleles. Enhancing rice cooking and eating quality is achieved through *Wx*^*b*^ and *ALK*^*TT*^ gene polymerization, while introducing the *fgr(E7)* gene significantly improved rice aroma. Using molecular marker-assisted technology, we aggregated these genes to develop a batch of *indica* hybrid rice parents with improved rice quality are obtained. Cross-combining these enhanced parents can generate new, high-quality hybrid rice varieties suitable for cultivation in Southern China. Therefore, our findings contribute to a molecular breeding model for grain quality improvement in high-quality indica hybrid rice. This study, along with others, highlights the potential of molecular design breeding for enhancing complex traits, particularly rice grain quality.

**Supplementary Information:**

The online version contains supplementary material available at 10.1186/s12284-024-00719-7.

## Introduction

Rice breeding aims to enhance yield and quality, its two crucial objectives. Heterosis utilization has significantly boosted rice yield in recent years. As living standards continue to rise, so does consumer demand for high-quality rice, making it essential for rice breeders to collaboratively improve yield and quality comprehensively (Xing and Zhang 2010; Harberd 2015).

Rice quality includes milling quality, appearance quality, eating and cooking quality (ECQ) and nutritional quality. Grain shape, chalkiness and transparency are important evaluation indexes of rice appearance quality. Cooking and eating quality are affected by amylose content (AC), gel consistency (GC), and gelatinization temperature (GT). High-quality rice typically exhibits long, thin grains, low chalkiness, medium AC, and strong aroma. Consequently, molecular design breeding efforts have primarily targeted grain shape, AC, GC, GT and aroma content to develop high-quality rice.

Southern China is the primary region for hybrid rice cultivation, but high temperatures during grain filling stages often compromise the production of high-quality rice (Smith et al. 1997; Preiss and Sivak 1998; Huang et al. 2013; Calingacion et al. 2014; Zhao et al. 2018).The pursuit of super-high yield rice has led to early elite hybrid rice combinations producing larger and wider grains, which are more challenging to fill compared to long and thin grains, thereby negatively affect grain quality (Gong et al. 2017). Consequently, most consumers from Southern China, India, Thailand, Vietnam, the Philippines, Malaysia, Indonesia, and Pakistan prefer longer and slender rice grains (Bai et al. 2010; Yang et al. 2023a), necessitating the rejuvenation of parental lines in some early elite hybrid rice combinations to meet current market demands through grain shape improvement. In the early stage, we conducted a genome-wide association study and allelic functional nucleotide polymorphisms analysis of quality trait genes revealed that *ALK*, *FGR1*, *FLO7*, *GL7/GW7*, *GLW7*, *GS2*, *GS3*, *ONAC129*, *OsGRF8*, *POW1*, *WCR1*, and *Wx* were associated with the genetic enhancement of rice quality traits in Southern China. An analysis of 13 crucial rice quality genes, including the fragrance gene *fgr*, indicated that only a few varieties, such as Gui516, Gui569, Gui721, Ryousi, Rsimiao, Rbasi, and Yuehui9802, possessed multiple superior alleles in Southern China (Yang et al. 2023b). Therefore, it is essential to incorporate high-quality genes in rice breeding programs to achieve high-quality trait aggregation.

Grain shape significantly influences the appearance and processing qualities of rice, particularly affecting chalkiness and transparency. For example, enhancing the grain length-width ratio, through increased grain length or reduced width, significantly improve grain chalkiness and transparency (Song et al. 2007; Hu et al. 2015; Gong et al. 2017). Although numerous genes or QTLs (Quantitative Trait Loci) associated with grain development have been identified, only a few major regulators of rice grain shape have been discovered. Key determinants of rice grain length include *GS3* (Fan et al. 2006), *qGL3* (Zhang et al. 2012) and *GLW7* (Si et al. 2016), while *GW2* (Song et al. 2007), *GW5* (Liu et al. 2017), *GS5* (Li et al. 2011) and *GW8* (Wang et al. 2012) primarily regulate grain width. Contrastingly, *GS2* (Hu et al. 2015), *TGW3* (Ying et al. 2018) and *GW7* (Wang et al. 2015) predominantly affect grain length-width ratio. Among these, *GS3* is a major gene affecting grain length and weight, with minor effects on grain width and plumpness. A mutation in the second exon of *GS3*, replacing the cysteine-encoding codon TGC with a premature stop codon TGA at position 55, results in the loss-of-function allele *gs3*, conferring a long-grain trait (Fan et al. 2006; Mao et al. 2010). Additionally, *GW8* (*OsSPL16*) encodes an SBP-domain transcription factor that positively regulates grain width, impacting grain size, shape, and quality. Both the deletion allele *gw8* and loss-of-function allele *gw8*^*Amol*^ produce particularly slender grains (Wang et al. 2012). Pyramiding *gw8* and *gs3* has been previously shown to significantly enhance the efficiency of rice quality breeding in previous studies (Wang et al. 2012; Dai et al. 2016). Another crucial positive regulator of rice grain length and appearance quality is *GW7* (Miura and Matsuoka 2015; Wang et al. 2015), which is directly repressed by GW8 through binding to its promoter. The semidominant *GW7*^TFA^ allele contains a mutation in the GW8 binding motif of *GW7* promoter, deregulating GW8’s repression and leading to increased *GW7* gene and slender grain production (Miura and Matsuoka 2015; Wang et al. 2015). Similarly, pyramiding *GW7*^TFA^ and *gs3* also produced particularly slender grains. However, despite the identification of numerous QTLs and genes controlling rice grain shape, the impact of various allele combinations on grain shape and their selection in the breeding process to achieve desired grain shapes remains largely unknown. The specific alleles of key grain shape genes, such as *GS3*, *GS5*, *GW5* and *GL7*, have been found to be utilized in Guangdong Simiao varieties, a popular kind of rice in Southern China, and selected for grain shape enhancement (Yang et al. 2023a).

The *Chalk5* gene significantly impacts the chalkiness and transparency of rice grains, influencing appearance quality, milled rice yield, and total protein content (Li et al. 2014). Down-regulation of *Chalk5* expression due to two SNPs at either − 485 or -721 sites in the promoter region significantly reduces endosperm chalkiness (Li et al. 2014). Therefore, introducing the low chalky allele *chalk5* can improve the chalky character of rice.

Three key factors, AC, GC, and GT, affect the eating and cooking qualities (ECQ) of rice grains (Singh et al. 2006). *Wx* gene regulates AC, with allelic variation closely correlated to differences in AC in rice endosperm (Wang et al. 1995; Cai et al. 1998; Tian et al. 2009). The two predominate alleles, *Wx*^*a*^ and *Wx*^*b*^ alleles correspond to high and medium-low AC, respectively (Sano 1984). The *ALK* gene encodes *Soluble Starch Synthesis gene IIa* (*SSIIa*), with allelic variation causing GT variation among rice varieties (Tian et al. 2009; Gao et al. 2011). The *ALK*^*c*^ allele controls high GT, while the *ALK*^*TT*^ allele confers low GT (Jeon et al. 2010). Additionally, *Wx* and *ALK* were major genes controlling GC in rice (Tian et al. 2009).

The increasing popularity of fragrant rice is due to its appealing aroma and high quality, which are associated with the 2-acetyl-1-pyrroline (2AP) concentration. The *fgr* gene, responsible for rice flavor, is identical to the *Badh2* gene that encodes a betaine aldehyde dehydrogenase. A specific allele, *Badh2-E7* harbors an 8 bp deletion and 3 bp mutation in the #7 exon of the *Badh2* gene, resulting in a non-functional betaine aldehyde dehydrogenase. Consequently, the metabolic pathway of the betaine aldehyde dehydrogenase substrate 2-AP is disrupted, leading to continuous accumulation of the aroma compound 2-AP and enhancing the aroma in both leaves and grains (Bradbury et al. 2005). The *Badh2-E7* allele is most closely associated with aroma traits (Bradbury et al. 2005; Kovach et al. 2009).

Many crop breeders are focusing on breaking through traditional genetic breeding bottlenecks and implementing more efficient rice molecular design breeding. In this study, we developed and validated PARMS markers for seven major rice quality-related genes: *GS3, GW7, GW8, Chalk5, Wx, ALK*, and *FGR*. These markers were used to genotype their allele variation genes in 214 rice varieties or parental lines. Phenotyping the traits relevant to these genes in the 69 parental lines of hybrid rice allowed for analysis of their interactions influencing rice grain quality. New insights into the molecular basis of high-quality hybrid rice were gained, leading to the development of molecular design breeding strategies for each of the 13 selected elite parental lines to rapidly improve grain quality. Several hybrid rice combinations derived from these improved parental lines exhibited superior grain quality. The developed PARMS markers, the new insights into grain quality control, and improved parental lines resulting from this study will greatly enhance future high-quality rice breeding programs.

## Result

### Development of PARMS Markers for the Major Quality-Control Genes, *GS3, GW7, GW8, Chalk5, Wx, ALK* and *FGR*

To accelerate genotyping and molecular marker-assisted (MAS) breeding for high-quality rice, we developed PARMS markers for seven major quality-control genes (*GS3, GW7, GW8, Chalk5, Wx, ALK*, and *FGR*), following the method described in the Materials and Methods section. Ten PARMS sets were designed to detect different alleles of these seven genes, with primer sequences and distinguishable SNPs listed in Supplementary Table S1. We validated these markers by genotyping the 214 rice varieties or parental lines from our breeding germplasm collection. Results in Supplementary Figure S1 and Supplementary Table S2 confirmed that all ten PARMS sets specifically differentiated the allelic variations of their target genes. For example, the *GW7*-90 PARMS set, which detects the A/G SNP of the *GW7* gene at the upstream − 90 bp position, clearly distinguished this SNP in 211 out of 214 rice varieties. Similarly, the *Chalk5-*a PARMS set, detecting the T/C SNP of the *Chalk5* gene at the upstream − 485 bp position, unambiguously discerned this SNP in 213 out of 214 rice varieties (Supplementary Table S2, Supplementary Figure S1). These findings demonstrated that the developed PARMS sets reliably and effectively differentiated allelic variations linked to the functional differentiation of these seven quality-control genes.

Genetic diversity analysis indicates that these alleles have been stably inherited through long-term natural selection. The high-quality related alleles of *gs3*, *GW7*, *gw8*, *chalk5*, *Wx*^*b*^, *ALK*^*TT*^, and *fgr* have gradually been utilized in production. The utilization of grain shape genes is mainly based on *gs3* (allele frequency of 0.83), while *GW7* (allele frequency of 0.02) and *gw8* (allele frequency of 0.08) are less commonly used. *Wx*^*b*^ (allele frequency 0.79) and *ALK*^*TT*^ (allele frequency 0.72) have also been widely used in high-quality breeding. The application of aroma related *fgr (E7)* allele (allele frequency of 0.2) in production is gradually increasing (Supplementary Table S3).

### Genotype-Phenotype Correlation Analyses among the 69 Selected Elite Parental Lines or Varieties

We genotyped 214 rice varieties or parental lines of hybrid rice for these seven quality control genes and selected 69 elite lines for further genotype-phenotype correlation analyses. These lines represent the backbone parents of *indica* rice varieties or *indica* hybrid rice widely cultivated either in the past or at present (Supplementary Table S4). We phenotyped the selected 69 lines for rice grain length and width, AC, ASV, GC, and 2-AP (aroma substance) content (Supplementary Table S4). Upon examining the allelic variation distribution of the seven quality control genes in these 69 elite lines, we observed that the superior *gs3* allele, which confers larger and longer rice grains, was widely used in the past in 53/69 (53 out of 69) lines (Supplementary Table S4). This predominance reflects the yield-oriented breeding efforts of the past. The *Wx*^*b*^ allele associated with medium-low AC content was also highly represented (45/69), highlighting the continuous focus on improving eating and cooking quality (ECQ) (Supplementary Table S4). The favorable alleles, *chalk5* (lower chalkiness) and *ALK*^*TT*^ (lower GT) were relatively unbiased in distribution, with 27/69 and 30/69 lines, respectively. However, the favored alleles *GW7*^*TFA*^ and *gw8*^*Amol*^, linked to longer and slender rice grains, and *fgr*, associated with rice aroma, were underrepresented, with only 7/69, 4/69, and 8/69 lines, respectively (Supplementary Table S4).

**Fig. 1:**
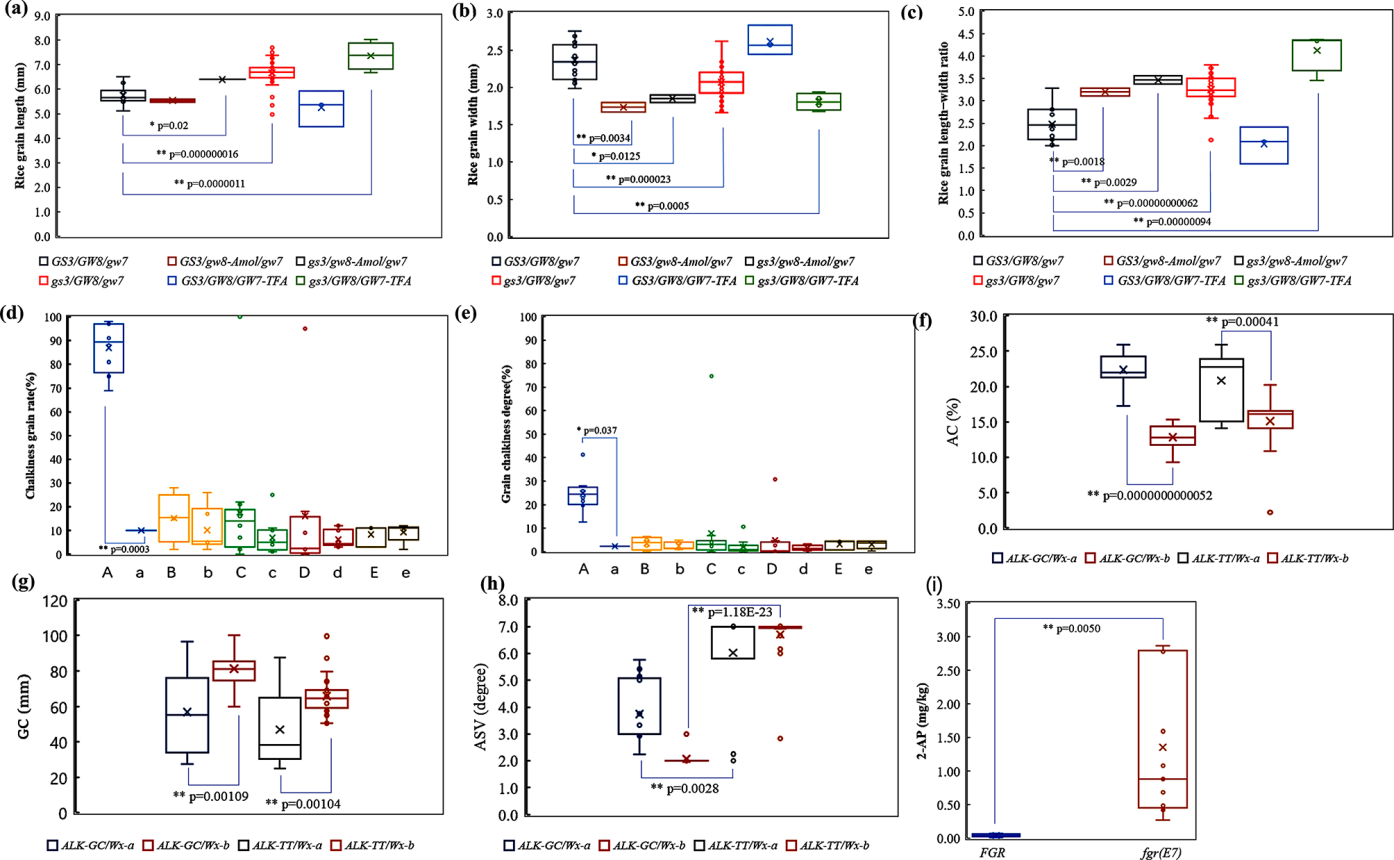
Effect analysis of the targeted genes genotype on rice quality. **(a)**, **(b)** and **(c)** Effects of the grain type genes *gs3*, *gw8* and *GW7* on grain length, grain width and grain length width ratio; **(d)** and **(e)** Effect of *Chalk5* and grain width on chalkiness. The abscissa is the grain width, A/a > 2.5 mm, B/b = 2.3 ~ 2.49 mm, C/c = 2.0 ~ 2.29 mm, D/d = 1.8 ~ 1.99 mm, E/e < 1.8 mm, Capital letters indicate the high chalkiness allele *Chalk5*, and lower case letters indicate the low chalkiness allele *chalk5*; **(f), (g)**, and **(h)** Effects of *Wx* and *ALK* on amylose content (AC), gel consistency (GC) and gelatinization temperature (GT) which was represented by alkali spreading value (ASV), respectively; **(i)** Effect of *fgr* on rice fragrance. Significant *Z test* was annotated in the graph, * *P* < 0.05, ** *P* < 0.01. The horizontal lines inside the box represent the median value. The upper side and lower side of the box represent the upper quartile and lower quartile, respectively. The whiskers represent the range of data, and small circles represent outliers

We categorized 69 elite lines into six groups based on the allelic variations of grain size and shape-related genes, *GS3*, *GW7*, and *GW8* (Fig. 1a-c): I (*GS3/GW8/gw7*, 15 lines), II (*GS3/gw8*^*Amol*^*/gw7*, two lines), III (*gs3/gw8*^*Amol*^*/gw7*, two lines), IV (*gs3/GW8/gw7*, 43 lines), V (*GS3/GW8/GW7*^*TFA*^, three lines), and VI (*gs3/GW8/GW7*^*TFA*^, four lines). The genotype-phenotype correlation analyses revealed that the *GS3* gene primarily controls grain length as lines with the *GS3* allele exhibited shorter grains than those with the *gs3* allele (I vs. IV and II vs. III, Fig. 1a). Pyramiding the *gs3* and *GW7*^*TFA*^ alleles further significantly increased grain length (V vs. VI and I vs. VI, Fig. 1a). The *gw8*^*Amol*^ allele played a major role in controlling grain width, as all lines carrying this allele displayed slender grains (II vs. I and III vs. IV, Fig. 1b). Additionally, group I (*GS3*/*GW8*/*gw7*) had wider grains than other group (I vs. II, III, IV, VI, Fig. 1b), indicating that *GS3* and *gw7* genes also contribute minimally to grain width control. Rice lines carrying the *gs3* and *GW7*^*TFA*^ alleles demonstrated increased grain length, while those with the major *gw8*^*Amol*^ allele and the minor *GW7*^*TFA*^ allele showed reduced grain width. Consequently, lines possessing either *gw8*^*Amol*^ or *GW7*^*TFA*^ had a higher grain length-width ratio (3.0-4.3) compared to those with the *GS3*/*GW8*/*gw7* (group I) genotype (2.0-2.9). Specifically, rice lines with the *gs3* and *GW7*^*TFA*^ allele combination (group VI) exhibited superior length-width ratios, surpassing 3.5 in the majority of these lines (Fig. 1c, Supplementary Table S4). This combination (group VI) consistently yielded favorable outcomes in grain length, grain width, and length-width ratio (Fig. 1a-c, Supplementary Table S4).

The degree and rate of chalkiness in polished rice grains significantly impact both grain appearance quality and ECQ, thus affecting market value. Chalkiness is primarily regulated by the *Chalk5* gene, highly correlated with grain width. Our analyses show that when grain width was > 2.5 mm, lines carrying the high chalkiness allele *Chalk5* produced grains with substantially higher chalkiness than those with the low chalkiness allele *chalk5*. For widths between 2.0 and 2.49 mm, the influence of *Chalk5* on chalkiness is weak, with smaller widths showing even weaker effects. The *chalk5* exhibited low correlation to chalkiness with grain widths below 2.0 mm, with minimal differences between *Chalk5* and *chalk5* alleles (Fig. 1d-e, Supplementary Table S4). We can also see from the correlation between the average effect of *GS3* alleles on grain shape and chalkiness that changes in grain shape greatly affect the formation of chalkiness (Supplementary Table S5). These findings suggest reducing grain width below less than 2.0 mm and is an effective strategy to mitigate chalkiness.

Consistent with previous studies (Wang et al. 1995; Cai et al. 1998; Tian et al. 2009; Jeon et al. 2010), we confirmed that the *Wx* gene primarily regulates the AC and GC of rice grains, while the *ALK* gene chiefly regulated the GT. The rice lines with the *Wx*^*a*^ allele produced grains with higher AC than those with the *Wx*^*b*^ allele. A strong negative correlation exists between AC and GC, where higher AC corresponds to lower GC and vice versa (Fig. 1f-g, Supplementary Table S4). The *ALK* gene significantly influences GT, with rice lines carrying the *ALK*^*TT*^ allele exhibiting lower GT than those with the *ALK*^*GC*^ allele, as represented by their ASV values. Interestingly, rice lines with the *ALK*^*GC*^ allele and the *Wx*^*b*^ combination displayed considerably lower ASV values than other lines for unknown reasons (Fig. 1h, Supplementary Table S4). Regarding rice aroma, the lines carrying the *fgr(E7)* allele contained significantly higher 2-AP content than those with the *FGR* allele (Fig. 1i, Supplementary Table S6).

A phylogenetic tree was constructed using the genotyping results of these 69 parental lines, which were clustered into three major branches. Through combined analysis with phenotype, it was found that high-quality materials are mainly concentrated in the III-1 branch of the third major branch. (Supplementary Figure S2). This further confirms that these alleles are closely related to the quality of rice.

### Improve the Grain Quality of Elite Parental Lines of Hybrid Rice by Molecular Design Breeding

Over the past few decades, rice breeders’ efforts have led to the development of elite varieties and parental lines of hybrid rice, significantly contributing to global food security. However, many of these varieties have become obsolete or have been withdrawn from the current market, primarily because their grain quality fails to meet current market preferences. Therefore, updating or improving these elite varieties and parental lines for the current market holds great value for rice breeding. Leveraging our newly acquired knowledge from genotype-phenotype correlation analyses, we aimed to enhance the grain quality of six cytoplastic male sterile (CMS) lines and seven restorer lines using the molecular design breeding strategy described in the Materials and Methods section. By pyramiding the superior alleles, including *gs3*, *GW7*^*TFA*^, *gw8*^*Amol*^, *Wx*^*b*^, *ALK*^*TT*^, *chalk5*, and *fgr(E7)*, we anticipate expediting the achievement our breeding objective: to develop high-yielding hybrid rice with premium quality.

**Fig. 2:**
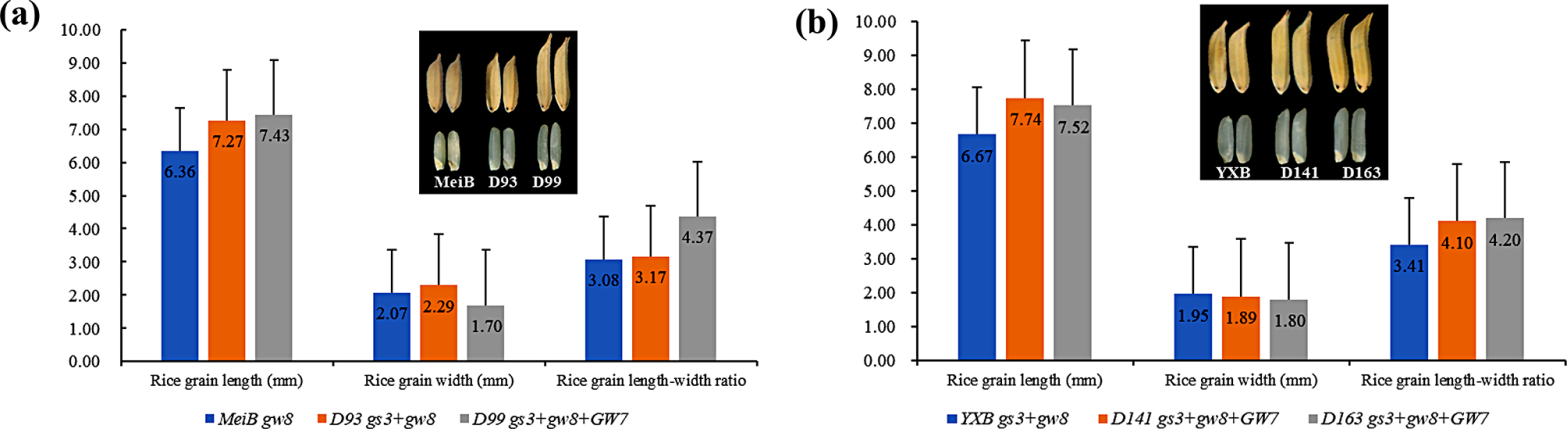
Effect of the grain type gene polymerization on appearance quality of rice. **(a)** Grain shape characteristics of MeiB and its improved lines D93 and D99; **(b)** Grain shape characteristics of YXB and its improved lines D141 and D163

MeiB, maintainer line of the elite CMS line MeiA with a slender grain phenotype due to carrying the *gw8*^*Amol*^ allele, has a grain length-width ratio of ~ 3.08. Introducing the larger and longer grain allele *gs3* resulted in the improved line D93, with significantly increased grain length and width. However, the combination of *gs3* and *gw8*^*Amol*^ led to a slight increase in the grain length-width ratio from 3.08 to 3.17, compared to the original MeiB. Another improved line D99 was developed by pyramiding four favorable alleles: *gs3*, *gw8*^*Amol*^, *GW7*^*TFA*^ and *fgr(E7)*. Compared to D93, D99 exhibited a significant decrease in grain width (from 2.29 mm to 1.70 mm) and a slightly longer grain length became a little longer (from 7.27 mm vs. 7.43 mm), resulting in a significant increase in the grain length-width ratio to 4.37, compared to MeiB’s 3.08 and D93’s 3.17 (Fig. 2a, Supplementary Table S7). Additionally, the *fgr(E7)* allele in the improved D99 substantially enhanced MeiB’s aroma (Supplementary Table S6). Similarly, the maintainer line YXB of the elite CMS line YXA carrying *gs3* and *gw8*^*Amol*^ alleles, was improved by adding the *GW7*^*TFA*^ allele, which resulted in two new improved lines, D141 and D163, with substantially increased grain length and length-width ratios (Fig. 2b, Supplementary Table S7).

**Fig. 3:**
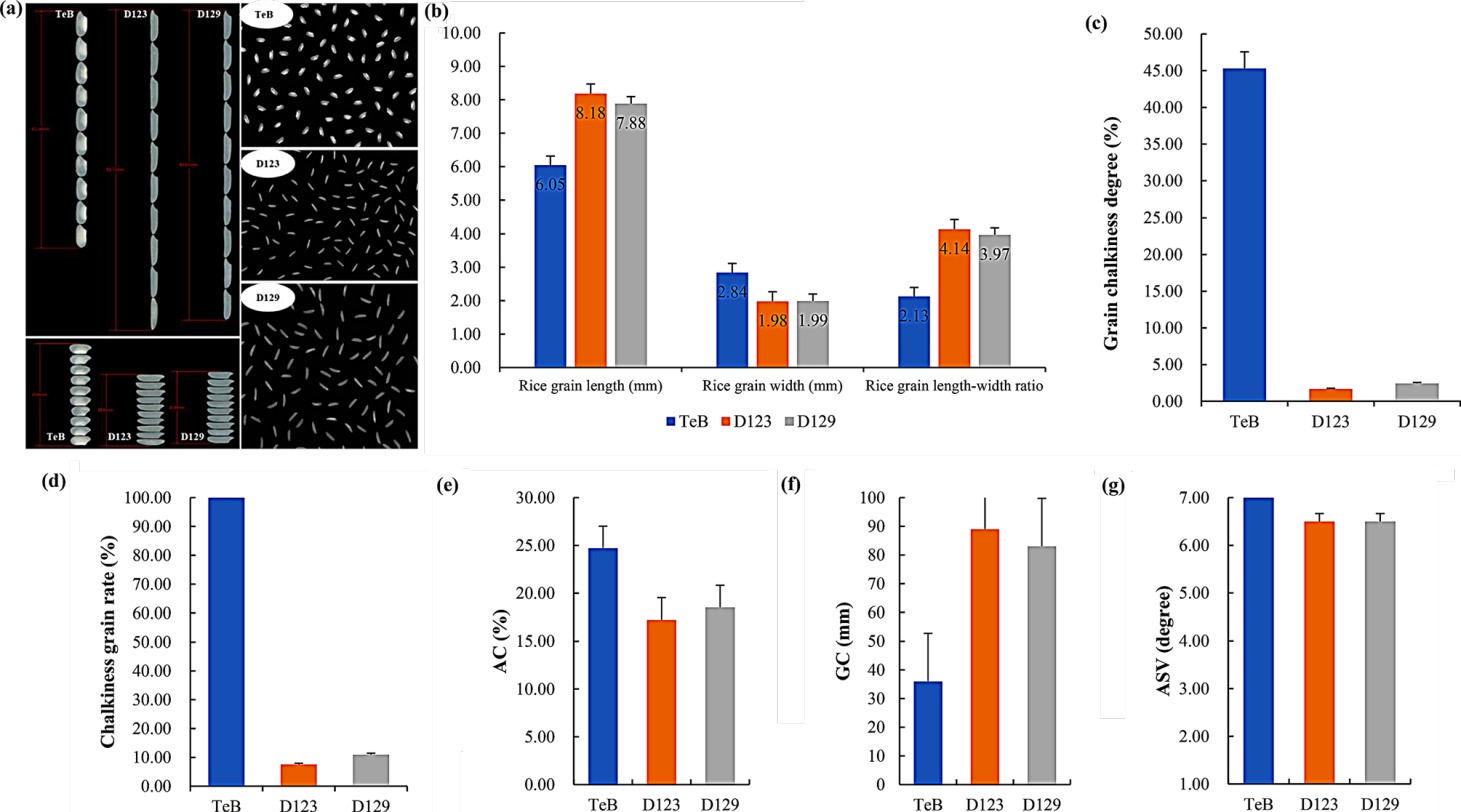
Enhancement of the grain quality in TeB by gene polymerization. **(a)** The milled grain performance of TeB and its improved lines D123 and D129; **(b)** Comparison of the rice grain length, grain width and length–width ratio in TeB and its improved lines D123 and D129; **(c)** and **(d)** Comparison of the chalkiness characteristics in TeB and its improved lines D123 and D129; **(e-g)** Comparison of the AC, GC and ASV in TeB and its improved lines D123 and D129, respectively

TeA is an elite CMS line, recognized for its large grain, exceptional plant architecture, and high yield potential, has resulted in its numerous high-yielding hybrid rice combinations being bred and widely cultivated in China. However, TeA’s short and wide grain, high chalkiness grain rate and grain chalkiness degree, and high AC rendered its hybrid rice unsuitable for the current market, TeA to become increasingly obsolete. To address these shortcomings, we employed the TFB donor line, rich in high grain quality genes, to enhance the grain quality of TeB (TeA’s maintainer line). By substituting TeB’s inferior *GS3*, *gw7*, and *Wx*^*a*^ alleles with TFB’s superior *gs3*, *GW7*^*TFA*^ and *Wx*^*b*^ alleles while preserving the superior *ALK*^*TT*^ allele of TeB, we developed two improved lines (D123 and D129). The rice quality parameters of both lines were significantly enhanced compared to TeB (Fig. 3a, Supplementary Table S6). Notably, the appearance quality improved, with D123 and D129 exhibiting increases in rice grain length by 35.38% and 30.42%, respectively, and decreases in rice grain width by 30.39% and 30.04%, respectively. Due to this grain length increase and the grain width decrease, the rice grain length-width ratio changed from 2.13 to 4.14 and 3.97 (Fig. 3b), respectively. Additionally, the grain chalkiness degree and chalkiness grain rate significantly reduced, with the grain chalkiness degree decreasing from 45.31 to 1.69% and 2.44% (Fig. 3c), and the chalky grain rate drastically dropping from 100 to 7.90% and 11.58% (Fig. 3d), respectively. Compared to the original TeB, the improved lines D123 and D129 exhibited substantial amelioration in ECQ-related AC and GC. AC decreased from 24.71 to 17.23% and 18.52% (Fig. 3e), while GC increased from 36 mm to 89 mm and 83 mm (Fig. 3f), respectively. Moreover, GT remained at or above 6.5 (Fig. 3g). These results demonstrated that pyramiding the superior gene alleles gs*3*, *GW7*^*TFA*^, *ALK*^*TT*^ and *Wx*^*b*^ sufficiently upgraded the grain quality of an obsolete elite parental line.

**Fig. 4:**
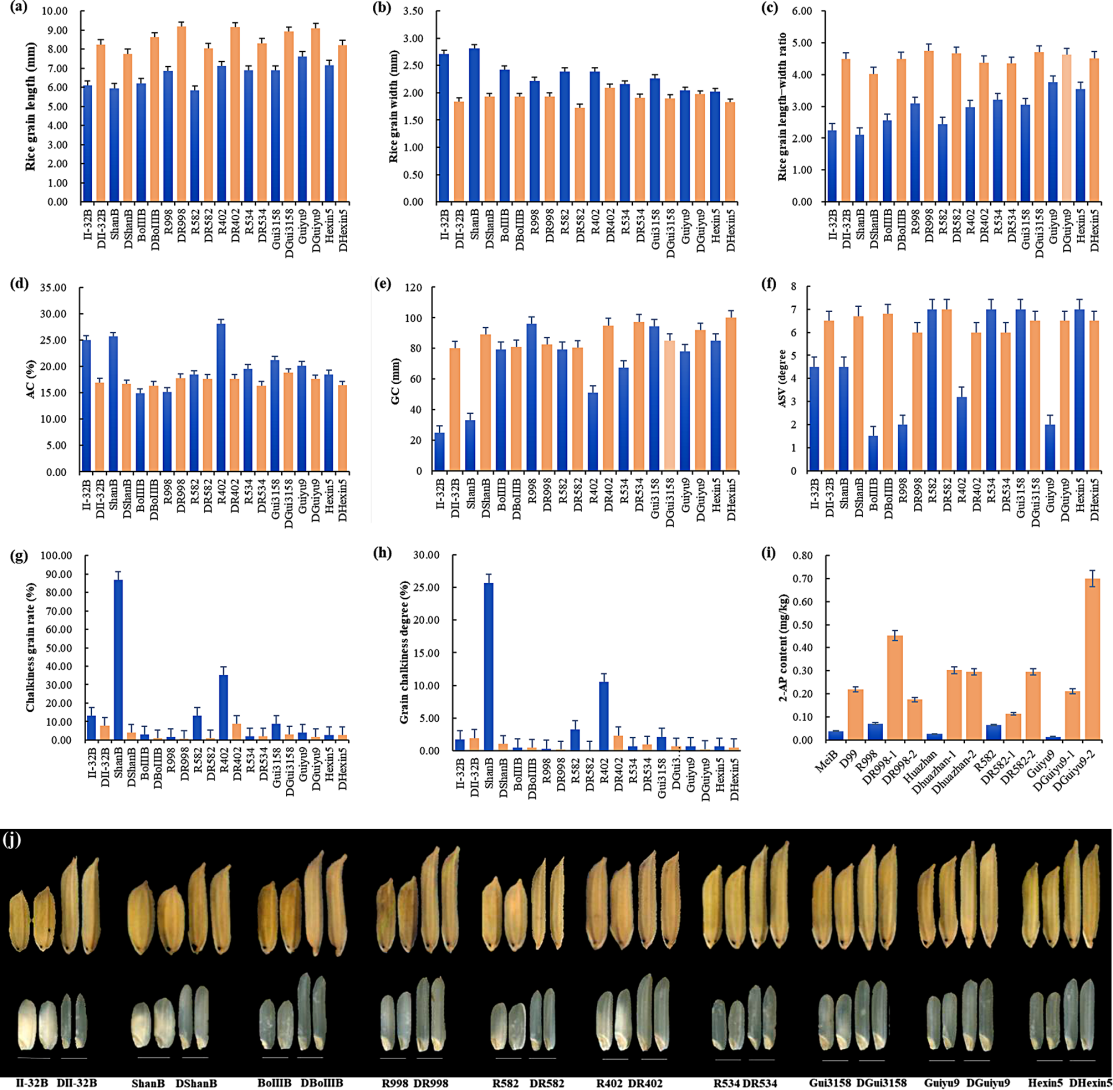
Enhancement of the grain quality in hybrid rice parents by gene polymerization breeding. **(a-c)** Comparison of the rice grain length, grain width and length–width ratio in parents and its improved lines (D letter head), respectively; **(d-f)** Comparison of the AC, GC and GT in parents and its improved lines, respectively; **(c)** and **(h)** Comparison of the chalkiness characteristics in parents and its improved lines; **(i)** Comparison of the 2-AP content in parents and its improved lines; **(j)** The grain performance of the parents and its improved lines

Besides the previously mentioned three improved CMS lines, another three CMS lines (ShanA, II-32A, and BoIIIA) and seven restorer (R) lines (R998, R582, R402, R534, Gui3158, Guiyu9, and Hexin5) were enhanced using the same strategy (Fig. 4, Supplementary Table S7). The data analysis of CMS lines utilizes their corresponding maintainer lines for statistical purposes. These improved parental lines exhibited significant enhancements in grain length, grain width, grain length-width ratio, chalkiness, AC, GC, and GT. The grain length, grain width, and length-width ratio of the improved lines ranged from 7.75 to 9.18 mm (Fig. 4a), 1.73 to 2.09 mm (Fig. 4b), and 4.03 to 4.75 (Fig. 4c), respectively. Their AC fell within the favorable medium range (16.29–18.76%) (Fig. 4d). Moreover, their GC (80–100 mm; Fig. 4e) and GT (ASV grade 6.0–7.0; Fig. 4f) met the standards of high-quality rice. Concurrently, introduction of the *fgr(E7)* gene also considerably improved the rice’s aroma (Fig. 4i, Supplementary Table S6). Therefore, our results demonstrated that pyramiding *gs3*, *GW7*, *gw8*, *ALK*^*TT*^, *chalk5*, *Wx*^*b*^, and *fgr(E7)* alleles represents a broadly applicable strategy for rice quality improvement (Fig. 4j).

### Improved Elite Parental Lines Significantly Increase the Possibility to Breed Hybrid Rice with Superior Quality

**Fig. 5:**
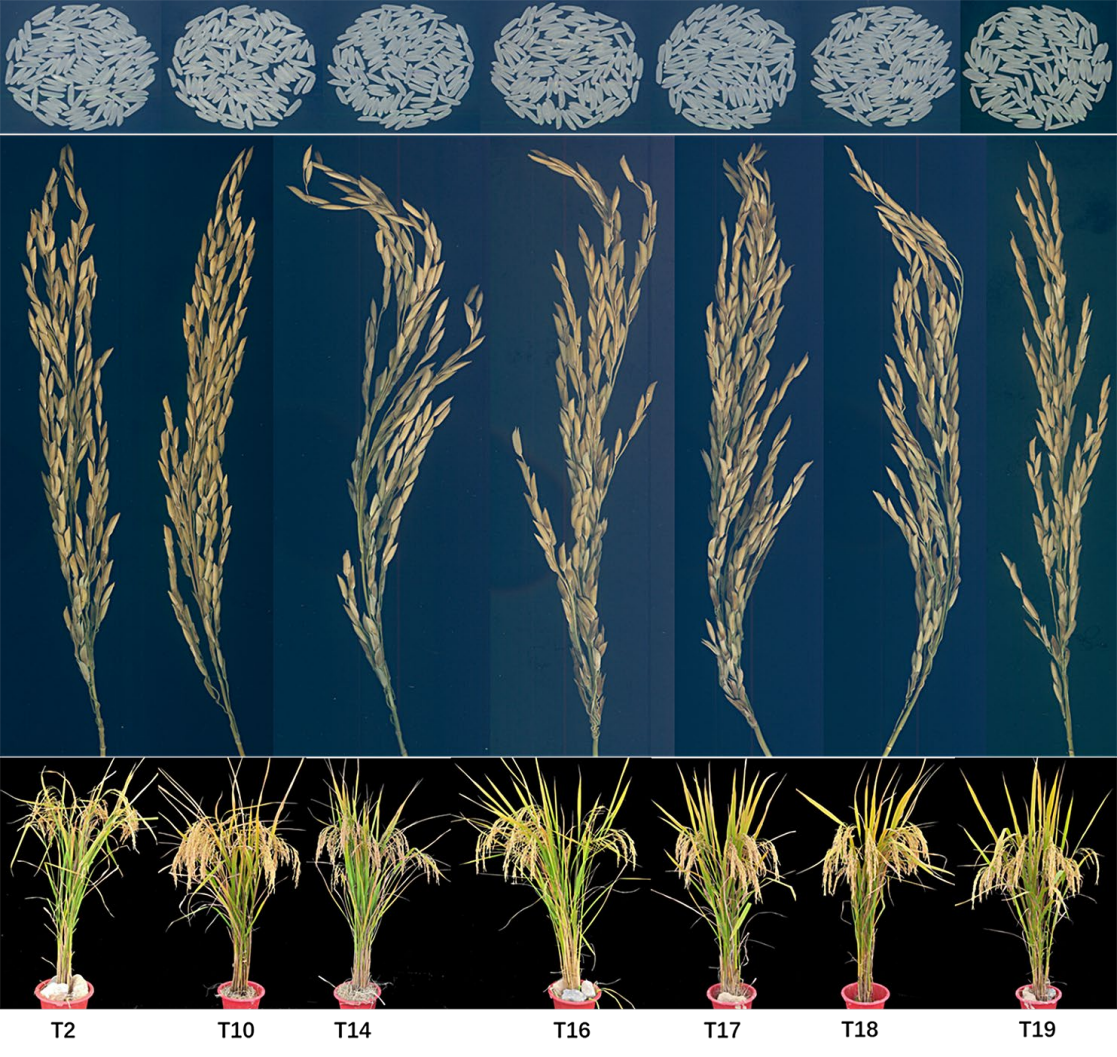
The combinations of hybrid rice with high quality. **(a)** The appearance of rice; **(b)** The characteristics of rice panicles; **(c)** The plant type characteristics of rice

To assess the potential of breeding superior quality hybrid rice using high-quality CMS lines and improved R lines, we performed crosses between them, resulting in nine hybrid rice combinations. Upon analyzing their rice quality, one hybrid rice combination met the premium quality grade 3, and six combinations achieved the premium quality grade 1 of the Chinese standard NY/T 593–2021 (Tang et al. 1999) for cooking rice variety quality (Fig. 5; Table 1). These findings demonstrate that utilizing parental lines enhanced in appearance quality and ECQ significantly increases our likelihood of breeding hybrid rice with superior quality. However, the slender grains of the rice hybrid combination had a negative impact on yield, the increase in grain length-width ratio resulted in a decrease in the thousand grain weight of the rice hybrid combination. The thousand grain weight of these superior quality combinations was generally less than 21 g (Table 1). The pursuit of a high grain length-width ratio would disrupt the balance between yield and quality. Therefore, to achieve both high-quality and high-yield goals in synergy, it is necessary to simultaneously consider multiple traits such as appropriate grain length-width ratio and effective grain number per spike.

**Table 1:** Rice quality analysis of high-quality hybrid rice combinations

Combination name	gs3	GW7	gw8	Chalk5	Wx	Alk	fgr	Rice grain length (mm)	Rice grain width (mm)	Rice grain length-width ratio	Chalkiness grain rate (%)	Grain chalkiness degree (%)	GC (mm)	ASV (degree)	AC (%)	2-AP content (mg/kg)	1000 grain weight (g)	Rice quality grade^2^
T2	*gs3*	H^1^	H	H	*Wx* ^*b*^	H	H	7.345	1.906	3.857	1.98	0.12	87.50	6	15.80	0.66	20.08	1
T10	*gs3*	H	H	*chalk5*	*Wx* ^*b*^	H	H	7.177	1.945	3.693	6.05	0.21	75.33	5	15.18	0.62	20	3
T12	*gs3*	H	*GW8*	H	H	H	*FGR*	7.13	2.13	3.35	3.62	0.40	84.33	3.39	15.65	Not detected	25.04	/
T13	*gs3*	H	*GW8*	H	*Wx* ^*b*^	H	*FGR*	7.37	2.18	3.39	2.62	0.27	71.00	3.61	18.01	Not detected	25.4	/
T14	*gs3*	*GW7*	*H*	*chalk5*	*Wx* ^*b*^	H	H	6.866	1.966	3.496	1.14	0.03	78.00	6	17.77	0.69	19.86	1
T16	*gs3*	*GW7*	*GW8*	*chalk5*	*Wx* ^*b*^	*Alk* ^*TT*^	*fgr(E7)*	7.208	1.768	4.084	5.79	0.30	74.50	7	16.20	2.28	18.81	1
T17	*gs3*	*GW7*	*GW8*	*chalk5*	*Wx* ^*b*^	*Alk* ^*TT*^	*fgr(E7)*	7.391	1.805	4.098	0.00	0.00	64.33	7	17.05	3.07	17.51	1
T18	*gs3*	*GW7*	*GW8*	*chalk5*	*Wx* ^*b*^	*Alk* ^*TT*^	*fgr(E7)*	7.337	1.802	4.077	3.08	0.16	63.67	7	17.80	2.45	18.42	1
T19	*gs3*	H	*GW8*	H	*Wx* ^*b*^	H	H	7.516	1.744	4.311	1.13	0.03	65.00	7	17.47	0.66	18.83	1

^1^ Heterozygous locus. ^2^ Base on the standard NY/T 593–2021 for cooking rice variety quality

## Discussion

Conventional breeding technology has been instrumental in enhancing rice yield over the past few decades but has certain limitations, such as long breeding cycles, high contingency, and low breeding efficiency. Traditional breeding methods for yield-related traits, like grain size and spike number, rely heavily on visual selection and breeders’ expertise developed over years of experience. easily distinguishable, making successful selection possible. However, traits such as grain ECQ, AC, and resistance to biotic and abiotic stress require laboratory tests or specific conditions for differentiation, making selection more difficult and inefficient, particularly in early segregating populations. Consequently, achieving a balance between yield and grain quality traits is difficult, with high-yielding varieties often exhibiting poor grain quality and good quality varieties having relatively lower grain yield. This issue is particularly prevalent in early hybrid rice, as its CMS parental lines typically have poor grain quality, and genetic improvement of these lines is a lengthy and arduous process. The problem of unbalanced yield and quality somehow restricts the production and promotion of hybrid rice. In contrast, molecular design breeding offers a solution by significantly increasing breeding efficiency and shortening breeding cycles through strategic planning. This approach enables accurate enhancement of current varieties’ shortcomings and rapid aggregation of multiple desirable traits. As more genes controlling crucial agronomic traits are identified and their roles in regulating complex traits are elucidated, precise molecular design breeding in rice is poised to advance rapidly. A recent study by Zeng et al. (Zeng et al. 2017) exemplifies this trend, demonstrating significant improvements in both yield and quality by pyramiding grain shape genes, such as *gs3* and *GW5* using the molecular design breeding strategy.

High temperature “forced ripening” frequently impairs indica hybrid rice production in Southern China, resulting in poor grain quality and high grain chalkiness. To address this issue, we aimed to improve the grain shape of parental lines of elite hybrid rice combinations using the molecular design breeding strategy, focusing on superior grain shape gene alleles *gs3*, *GW7*^*TFA*^, and *gw8*^*Amol*^. Additionally, we sought to enhance overall grain quality by targeting four other superior alleles: *Wx*^*b*^, *ALK*^*TT*^, *chalk5*, and *fgr(E7)*, associated with medium to low AC, low GT, low chalkiness and fragrance, respectively. Our study comprised four steps to achieve these goals and validate our approach. First, we developed PARMS markers for each of the seven targeted gene alleles (Supplementary Table S2, Figure S2). These markers were validated using 214 rice varieties from our core germplasm, accurately differentiating allele variations for all seven targeted genes, such as *Wx*^*a*^*/Wx*^*b*^, *GS3/gs3, GW7*^*TFA*^*/gw7, ALK*^*CC*^*/ALK*^*TT*^, *Chalk5/chalk5* and *FGR/fgr(E7)* (Supplementary Figure S1, Table S4). Subsequently, these PARMS markers were employed in late recurrent selection processes to identify individual plants carrying the targeted alleles (Supplementary Table S6, S7, Figure S1). The PARMS markers employed in this study reliably and sufficiently discriminated the allelic variations of these seven targeted genes, thereby proving to be valuable tools for rice quality improvement by other breeders. We genotyped and phenotyped 69 rice varieties from our core germplasm (Fig. 1, Supplementary Table S4), subsequently performing genotype-phenotype correlation analyses. These analyses offered novel insights into the gene interactions determining rice grain quality and the relative contributions of each gene to quality traits. For example, the *chalk5* gene emerged as the major regulator of grain chalkiness. Our findings indicated that the superior *chalk5* allele, which primarily contributes to low grain chalkiness, significantly reduced grain chalkiness only in rice lines with grain widths exceeding 1.8 mm. Consequently, which means when a rice line’s grain width is < 1.8 mm, the presence of either the superior *chalk5* or an inferior *Chalk5* allele will not substantially impact grain chalkiness (Fig. 1d-e, Supplementary Table S4). The alleles, *GW7*^*TFA*^ and *gw8*^*Amol*^, favoring longer and slender rice grains, along with the aroma linked *fgr* allele, were significantly underrepresented in these 69 elite lines (Fig. 1a-c and i, Supplementary Table S4), indicating ample opportunity for rice quality improvement using these superior *GW7*^*TFA*^ and *gw8*^*Amol*^ alleles. However, in fact, although *gw8* plays an important role in high-quality improvement, its impact on yield is also significant, which should be one of the reasons limiting its widespread application in rice breeding. Therefore, in high-quality breeding that focuses on yield, attention should be paid to the influence of *gw8* in the variety and careful utilization. Utilizing our newfound insights, we developed molecular design breeding strategies tailored to each of the six elite CMS maintainer lines and seven elite restorer lines to enhance grain quality (Supplementary Table S7). The resulting data demonstrated substantial improvement in the overall grain quality of all 13 elite lines (Supplementary Table S7, Figs. 2, 3 and 4). Furthermore, we created nine hybrid rice combinations using some of the improved CMS and restorer lines to assess their potential for producing premium-quality hybrid rice. Six out of the nine hybrid rice combinations achieved grade 1 premium quality (Table 1; Fig. 5).

Therefore, our study achieved its intended goals, demonstrating that the negative effects of high temperatures in Southern China on *indica* hybrid rice grain filling could be largely mitigated. This was accomplished by selecting rice grains with a length > 7.0 mm, a width < 1.8 mm, and a length-width ratio > 4.0, which resulted in minimal chalkiness. We successfully bred a series of parental lines for three-line hybrid rice with superior quality, and their hybrid combinations show great potential for improving rice grain quality. Prior to our improvements, the 13 parents selected in this study had been the primary sources of hybrid rice varieties in production, and exhibited excellent specific and general combining abilities for many years. Despite their poor quality, their hybrid rice varieties displayed outstanding yield performance and broad adaptability to various planting conditions. Therefore, further testing is required to determine whether these improved lines retained their exceptional characteristics.

During high-quality breeding, we encountered new challenges associated with the introduction of slender grain type genes. This genetic modification resulted in thinner and softer stems, as well as increased plant height in many improved lines, consequently reducing their lodging resistance. Additionally, slender grains often possess lower thousand grain weight compared to wider grains, potentially decreasing the yield of some varieties. Therefore, these drawbacks may impact their production application. Nevertheless, subsequent pyramiding of excellent alleles for lodging resistance and yield can address these issues.

## Conclusion

In conclusion, we found that the application of quality related alleles (*gs3*, *GW7*^*TFA*^, *gw8*, *chalk5*, *Wx*^*b*^, *ALK*^*TT*^, and *fgr*) significantly improved rice quality. Based on these findings, a molecular breeding model for high-quality *indica* hybrid rice grain qualities has been proposed. The potential of molecular design breeding for enhancing complex traits, particularly rice grain quality, is underscored by this study and others. The development of PARMS markers for seven high-quality genes, the adoption of a strategic methodology, and the acquisition of premium-quality parental lines provide a solid foundation for future rice grain quality improvement efforts.

## Materials and methods

### Development of the PARMS Markers

The tetra-primer amplification refractory mutation system serves (Ye et al. 2001) as the foundation for the penta-primer amplification refractory mutation system (PARMS), a simple and rapid SNP genotyping technology. In this technique, five primers, including a pair of universal fluorescent primers, allele-specific primer pairs, and a shared reverse primers were used to amplify SNP or short indel loci with allele specificity. Subsequently, fluorescence scanning was utilized for genotyping (Zhang et al. 2019; Jun et al. 2020).

PARMS markers specific for seven rice quality-related genes (*GS3, GW7, GW8, Chalk5, Wx, ALK*, and *FGR*) were developed using allelic variation information of those seven genes gathered from literature. SNPs were identified using BLAST and sequence alignment, and primer sets were designed employing Primer3Plus (https://primer3plus.com). The PARMS marker sequences and corresponding SNP details are listed in Supplementary Table S1. PCR amplification and genotype assays were performed following established protocols (Gao et al. 2021).

### Plant Materials

The developed PARMS markers were then used to genotype 214 *indica* hybrid rice parental lines and inbred varieties (Supplementary Table S2). Subsequently, 69 were selected for further genotype distribution and genotype-phenotype correlation analyses. These included 16 CMS maintainer lines, 23 CMS restorer lines, and 28 inbred varieties (Supplementary Table S4). For molecular breeding improvement, 13 lines were chosen: six CMS lines TeA, MeiA, YXA, ShanA, II-32 A, BoIIIA and seven restorer lines (R998, R582, R402, R534, Gui3158, Guiyu9, Hexin5) (Supplementary Table S7). All experimental rice plants were cultivated at the Guangxi Academy of Agricultural Sciences farm in Nanning (108°22′ E, 22°48′ N) during regular rice planting seasons.

### Measurements of the Grain Quality

Evaluation of the grain width, grain length, length-width ratio and chalky kernels was performed according to the National Standards of the People’s Republic of China (GB/T17891-1999) (Tang et al. 1999). Mature and dried seeds were shelled, and 100 randomly selected brown rice grains were scanned using a SC-G Automatic Seed Test and 1000-grain Weight Analyzer (Wanshen Testing Technology Co., LTD, Hangzhou, China) to measure the grain length, width, and length-width ratio. The average values were calculated from these 100 grains, with the length-width ratio determined as the grain length divided by its width. Additionally, 100 fully filled polished rice grains were randomly selected to assess grain chalkiness and chalkiness rate using a MICROTEH Scanner (MRS-9600TFU2L) and a Wanshen SC-E Rice Appearance Quality Analyzer (Wanshen Testing Technology Co., LTD, Hangzhou, China).

Mature rice grains were harvested, air-dried, and stored at room temperature for three months. For ECQ evaluation, ~ 150 g of grains were de-husked using a huller (SDL-A; CNRRI, Hangzhou, China) and milled using a JMJ-100 rice miller (CNRRI, Hangzhou, China). The ECQ parameters, including AC, GC, GT (evaluated as alkaline spreading value, ASV) were measured according to the methods reported previously (Huang et al. 2013). The grain ECQ parameter was analyzed independently in two consecutive years with three technical repeats for each test.

### Determination of the Volatile Compound 2-acetyl-1-pyrroline (2-AP) Content

The headspace solid phase microextraction (HS-SPME) method was employed to extract the total volatile components of rice (Kataoka et al. 2000). Refined rice, retrieved from a -80 °C refrigerator, was swiftly ground into refined rice powder. Then 3 g of the refined rice powder was transferred into a 15 ml headspace bottle, followed by complete sealing and insertion of an extraction head into the headspace bottle. Extraction was conducted in a water bath at 60 ℃ for 45 min with the headspace bottle containing the extraction head. Subsequently, gas chromatography-mass spectrometry (GC-MS) (Agilent 7890B-5977 A) was used to analyze the contents of 2-AP and other volatile components at 220 °C using the external standard method.

### Rational Design Breeding Procedure

Six elite CMS maintainer lines (B lines) and seven elite restorer lines (R lines) of three-line hybrid rice were selected to advance quality improvement. Those lines were used as the recurrent parents, while the elite CMS maintainer line TFB (carrying the favored *gs3*, *GW7*^*TFA*^, *ALK*^*TT*^ and *Wx*^*b*^ alleles) and the elite R line GX204 (carrying the favored *gs3*, *GW7*^*TFA*^, *ALK*^*TT*^, *Wx*^*b*^ and *fgr(E7)* alleles) were chosen as the donor parents for the B lines and R lines, respectively. Crossing, genotyping, selection, and backcrossing were performed from 2015 to 2021 as shown in Supplementary Figure S3. Using molecular marker assisted selection technology combined with backcrossing to introduce high-quality related alleles, each generation selects high-quality strains, and the higher generation self-crosses with homozygous genetic background to breed stable genetic single plants and form high-quality strains, such as MeiB and YXB (Supplementary Figure S4). Subsequently, the improved B lines with stable inheritance of agronomic traits were converted to corresponding CMS lines (A lines). The resulting high-quality A lines were then tested with high-quality R lines including the improved R lines, to breed premium quality hybrid rice.

### Data Analysis

Box-plot combinations is depicted by the genotypic and phenotypic data of 69 elite lines (Supplementary Table S4) in Microsoft’s Excel software. Significance testing be treatment by the *Z test* module and compare between every group. Basic statistics of genetic diversity including total number of alleles, and polymorphism information content (PIC) at each allele locus according to the formula PIC = 1-∑pi2 (Nei 1973). Display of phylogenetic tree using ggtree package in R language.

### Electronic Supplementary Material

Below is the link to the electronic supplementary material.

Supplementary Material 1

Supplementary Material 2

## Data Availability

All datasets generated for this study are included in the article/Supplementary file.
