# Supplementary material for: Genetic Effects of Grain Quality Enhancement in Indica Hybrid Rice: Insights for Molecular Design Breeding
Source: Rice (N Y). 2024 Jun 14;17:39. doi: 10.1186/s12284-024-00719-7 (PMC11178727; doi:10.1186/s12284-024-00719-7)
Supplement: Supplementary file 1 — Supplementary Material 1 [file 12284_2024_719_MOESM1_ESM.docx]

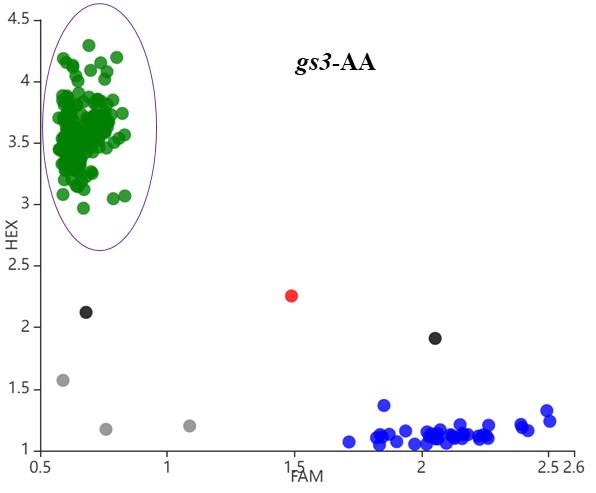

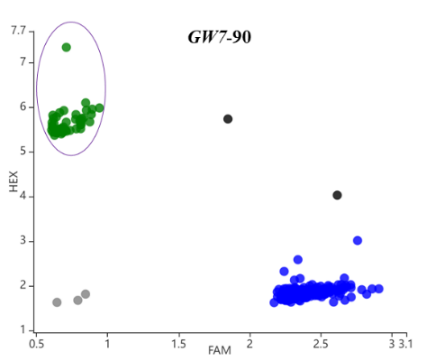

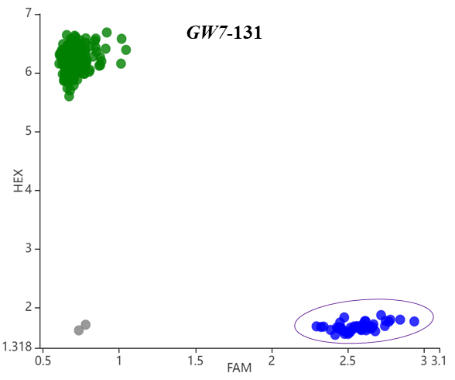

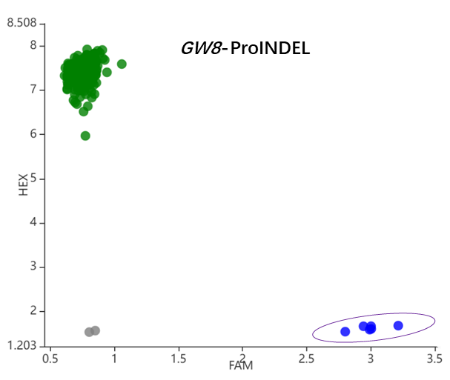

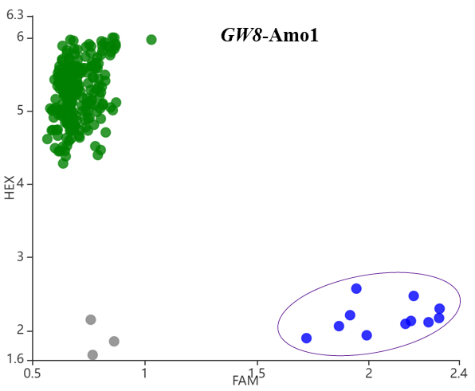

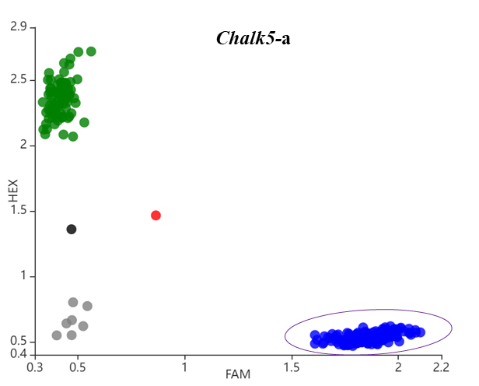

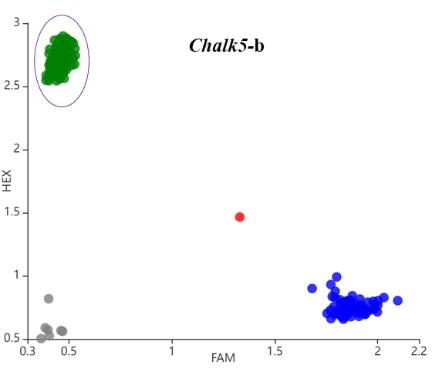

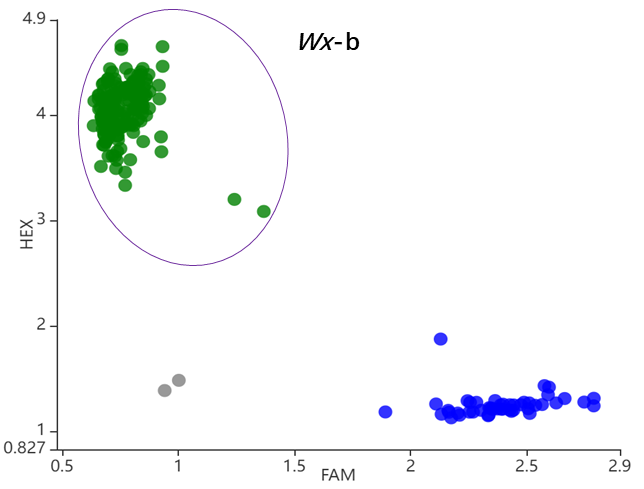

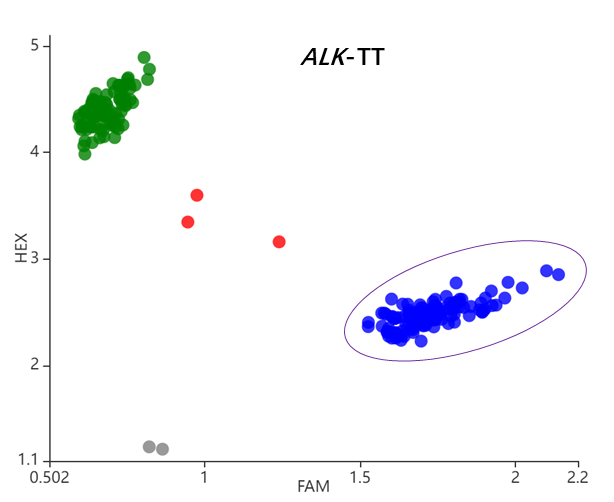

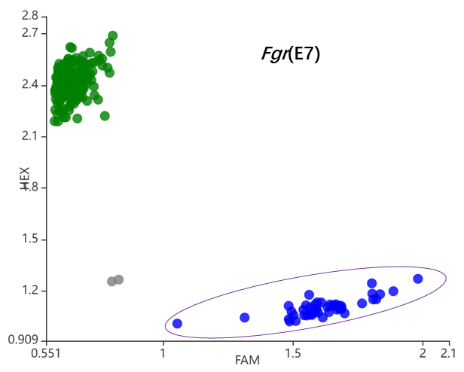


**Supplementary Figure S1.** **Development of functional molecular markers of targeted genes.** Excellent allele genotyping is shown in purple circles.


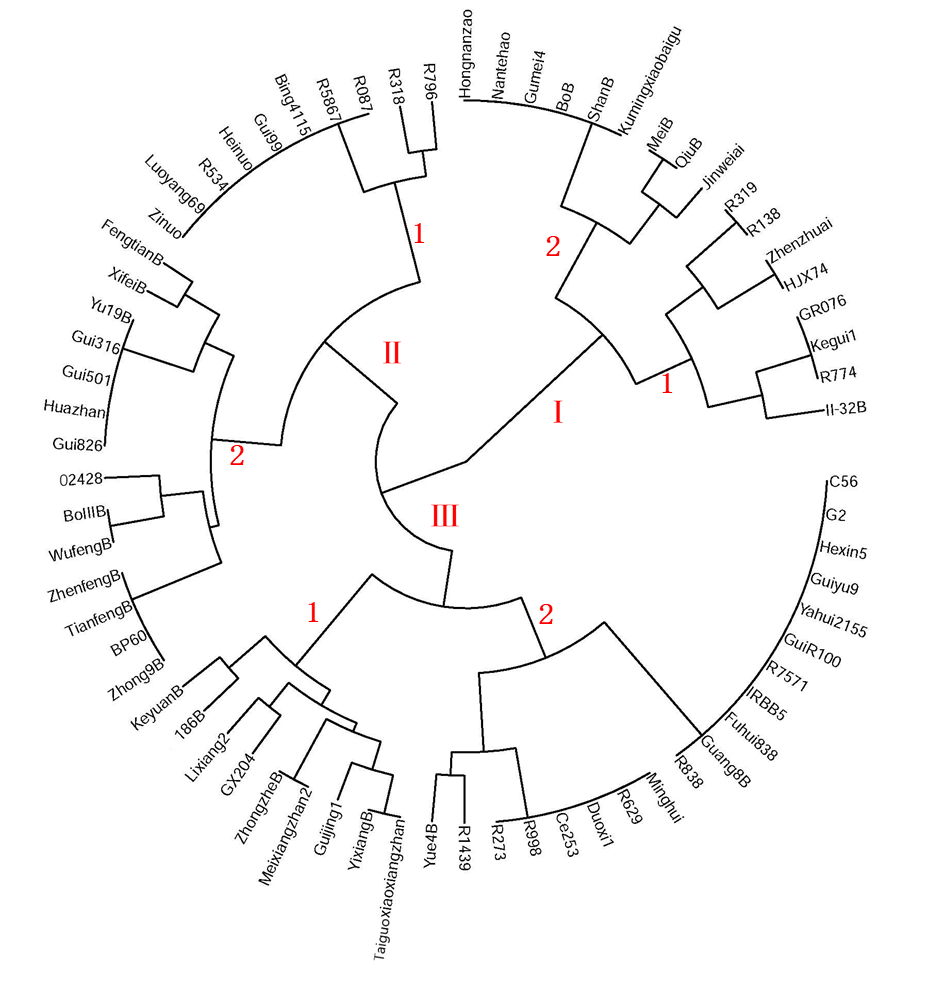


**Supplementary Figure S2.** Phylogenetic tree of these 69 parental lines. Symbols I, II, and III represent the three major branches of the phylogenetic tree, and numbers 1 and 2 represent the small branches on each major branch


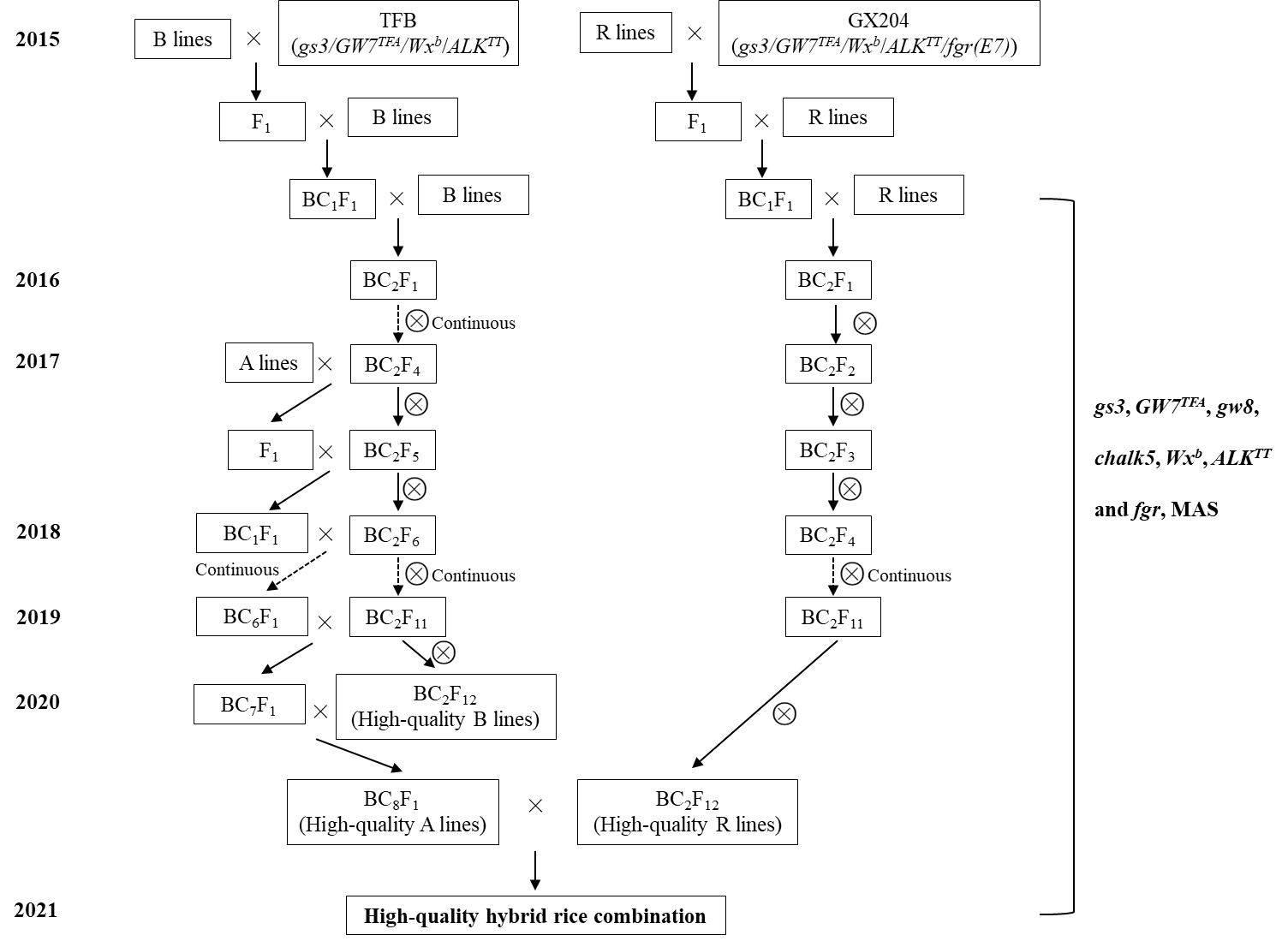


**Supplementary Figure S3.** **Molecular design breeding schedule.** The maintainer lines (B lines) and restorer lines (R lines) of CMS lines were selected as the recurrent parents, TFB and V204 were chosen as donor parents for B lines and R lines of CMS lines, respectively.


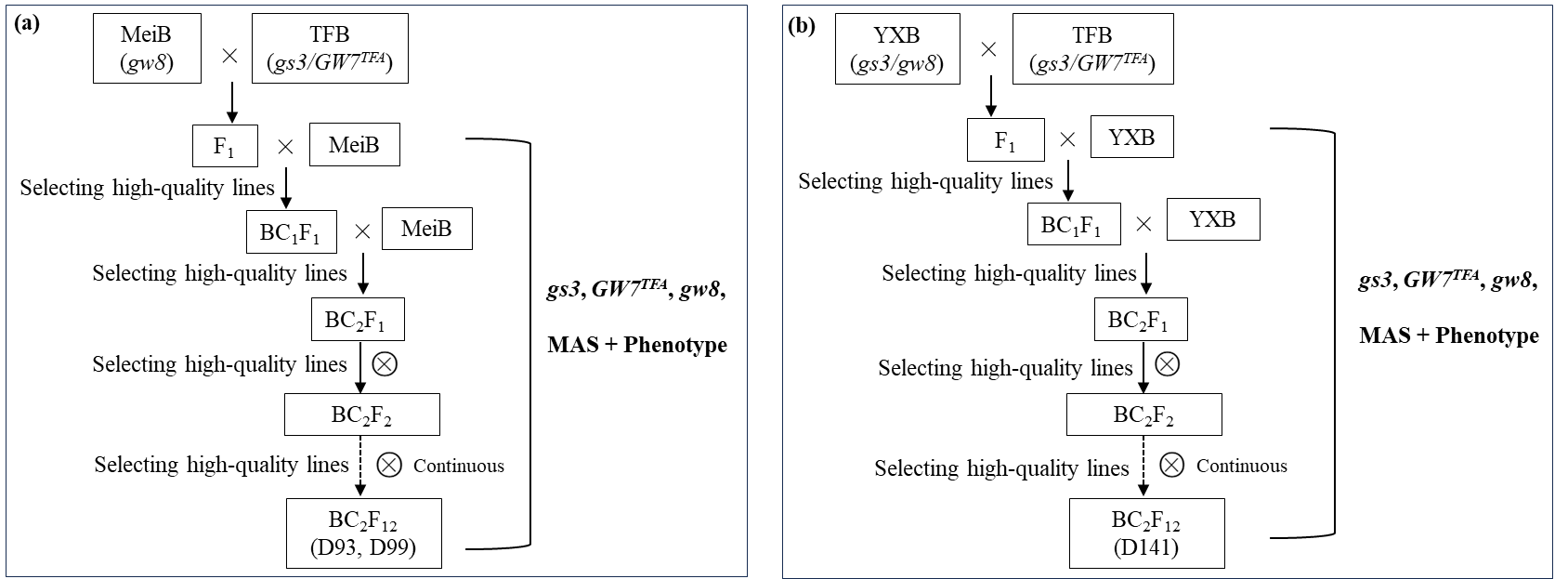


**Supplementary Figure S4.** **The breeding flow chart for improving grain shape in MeiB and YXB.** MeiB and YXB were selected as the recurrent parents, TFB was chosen as donor parents.
